# Biofilm imaging in porous media by laboratory X-Ray tomography: Combining a non-destructive contrast agent with propagation-based phase-contrast imaging tools

**DOI:** 10.1371/journal.pone.0180374

**Published:** 2017-07-21

**Authors:** Maxence Carrel, Mario A. Beltran, Verónica L. Morales, Nicolas Derlon, Eberhard Morgenroth, Rolf Kaufmann, Markus Holzner

**Affiliations:** 1 Institute of Environmental Engineering, ETH Zürich, Stefano Franscini-Platz 5, 8093 Zurich, Switzerland; 2 Swiss Federal Laboratories for Materials Science and Technology (EMPA), Dübendorf, Switzerland; 3 Department of Civil and Environmental Engineering, University of California Davis, Davis, California, United States of America; 4 Swiss Federal Institute of Aquatic Science and Technology (EAWAG), Dübendorf, Switzerland; Public Library of Science, UNITED STATES

## Abstract

X-ray tomography is a powerful tool giving access to the morphology of biofilms, in 3D porous media, at the mesoscale. Due to the high water content of biofilms, the attenuation coefficient of biofilms and water are very close, hindering the distinction between biofilms and water without the use of contrast agents. Until now, the use of contrast agents such as barium sulfate, silver-coated micro-particles or 1-chloronaphtalene added to the liquid phase allowed imaging the biofilm 3D morphology. However, these contrast agents are not passive and potentially interact with the biofilm when injected into the sample. Here, we use a natural inorganic compound, namely iron sulfate, as a contrast agent progressively bounded in dilute or colloidal form into the EPS matrix during biofilm growth. By combining a very long source-to-detector distance on a X-ray laboratory source with a Lorentzian filter implemented prior to tomographic reconstruction, we substantially increase the contrast between the biofilm and the surrounding liquid, which allows revealing the 3D biofilm morphology. A comparison of this new method with the method proposed by Davit et al (Davit et al., 2011), which uses barium sulfate as a contrast agent to mark the liquid phase was performed. Quantitative evaluations between the methods revealed substantial differences for the volumetric fractions obtained from both methods. Namely, contrast agent—biofilm interactions (e.g. biofilm detachment) occurring during barium sulfate injection caused a reduction of the biofilm volumetric fraction of more than 50% and displacement of biofilm patches elsewhere in the column. Two key advantages of the newly proposed method are that passive addition of iron sulfate maintains the integrity of the biofilm prior to imaging, and that the biofilm itself is marked by the contrast agent, rather than the liquid phase as in other available methods. The iron sulfate method presented can be applied to understand biofilm development and bioclogging mechanisms in porous materials and the obtained biofilm morphology could be an ideal basis for 3D numerical calculations of hydrodynamic conditions to investigate biofilm-flow coupling.

## Introduction

Biofilms are ubiquitous sessile microorganisms embedded in a self-produced matrix consisting of extracellular polymeric substances (EPS) [1]. The EPS matrix protects biofilms from their environment, so that they persistently develop and survive in industrial, natural or biomedical settings [2]. In water saturated soils, most microorganisms develop sessile lifestyles [3]. Biofilms are of high interest in this context, because of their natural contribution to the bioremediation of aquifers [4] or to reactive barriers [5], to microbial enhanced oil recovery [6] or to the sequestration of carbon dioxide [7, 8]. However, the growth of biofilms in porous media and the consequent bioclogging of the pore spaces [9] can also be detrimental as it can lead to the clogging of groundwater recharge wells [10] or deep geothermal systems [11]. It can also lead to an enhanced non-Fickian spreading of solute contaminants in groundwater [12], substantially complexifying the modelling and upscaling of mass transport in these systems [13–18].

Biofilms have been described as microbial landscapes [19] stretching over a large range of spatial scales [20] ranging from the micro- (individual cells), to the meso- (biofilm patches scale) or to the macroscale (reactor, aquifer scales). Accordingly, different experimental methods have been developed to investigate processes at different scales, from optical or confocal microscopes to characterize microscale development of biofilms in flow cells [21] or microfluidic devices [22], to studies using soil columns [23, 24] and characterizing the influence of biofilms on bulk system (macro-scale) properties (permeability reduction, dispersion, degradation rates of solutes etc.).

The mesoscale, which is the scale of interest in this study, is the scale where the biofilm structure is being shaped by the interplay of the hydromechanical and mass transfer processes. In porous media, this scale approximately corresponds to the pore-scale. Many investigations for porous media at that scale were performed using optical systems [25–30], but were limited to 2 dimensions. Lately, Optical Coherence Tomography (OCT) [31–33] stood out as a very powerful method to investigate these processes at the mesoscale for many different systems. However, OCT uses low coherent light in the visible range and the opacity of 3D samples limits the penetration depth. Therefore, OCT cannot be used for 3D porous media samples. Magnetic Resonance Imaging (MRI) is a great tool to obtain both flow and biofilm stuctural information, it is limited in spatial resolution (generally resolution coarser than 50 *μm*) which makes it difficult to access the exact liquid and biofilm phases from the transverse relaxation times (T2) [12, 34–36].

In the last few decades, X-ray microtomography (X-ray *μ*CT) became a standard tool for imaging soil samples [37, 38] and to image biofilm structures in 3D soil-like samples. Differentiating the biofilm from the liquid phase is a difficult task when using X-ray *μ*CT due to the high water content of biofilms and the consequently very close X-ray attenuation coefficients of water and biofilms. As a result, it is common to use chemical agents to increase the contrast between the biofilm and liquid phases. Particulate barium sulfate (BaSO_4_) suspensions were used as contrast agent to label non-biofilm colonized pores and imaged biofilm on a X-ray lab source [39]. Silver-coated 10 *μm* microspheres deposited at the biofilm surface revealed the biofilm-liquid interface using synchrotron radiation [40]. Another approach suggested was based on the use of 1-chloronaphtalene, an immiscible liquid with water, as a contrast agent [41, 42]. Finally, numerical pore-scale biofilm growth modeling was performed based on biofilm structures obtained at different Reynolds numbers from X-ray synchrotron tomography using BaSO_4_ as a contrast agent [43].

All the described methods based on X-ray *μ*CT to image biofilms either used a particulate suspension or a chemical to mark the liquid phase that was introduced *a posteriori* in the biofilm containing sample. The rationales behind the approach using particulate BaSO_4_ suspensions is first, that the BaSO_4_ particles are micrometer-sized and behave passively. Second, that the biofilm inner channels are smaller than the micrometer-sized barium suflate particles [44] and therefore, that advection of the BaSO_4_ particles within the biofilm is negligible. However, these rationales remind largely untested so far. Additionally, given that BaSO_4_ is a relatively heavy compound (*ρ*_*BaSO*_4__ = 3.62*g*/*cm*^3^), sedimentation issues arise, which can be exacerbated by the aggregation of the particles and then lead to motion blur artifacts. The use of additives such as xantham gum stabilizes these solutions but also change the rheological properties, potentially inducing biofilm detachment [39]. On the other hand, the approach based on silver-coated microspheres [40] suffered from the heterogeneous distribution of the silver-coated microspheres and possible interactions between the dense microspheres suspensions and the biofilm were not investigated. The 1-chloronaphtalene used as a contrast enhancing agent [41, 42] also has significant drawbacks, since this liquid is immiscible with water. Therefore, the non-wetting phase curvatures and contact angles may not contour exactly the interface with the aqueous biofilm phase. Additionally, it is not guaranteed that the capillary pressure requirements to invade all unclogged pores are met. Finally, 1-chloronaphtalene is a powerful pesticide whose interactions with biofilms have not been investigated yet.

In this study, instead of adding a potentially destructive contrast agent to the liquid phase, we use iron sulfate (FeSO_4_), a non-toxic inorganic compound naturally present in soils. FeSO_4_ is commonly used in biofilm studies [32, 45, 46]. The rationale behind this approach is that some biofilms naturally exhibit high content of inorganic matter (e.g. mineral precipitates [47]). FeSO_4_ is a compound naturally present in some aquifers and it can even be artificially introduced to enhance the bioremediation of contaminants (arsenic, uranium) by enhancing their precipitation [48, 49]. Here, the biofilm is cultured under the continuous addition of a solution containing FeSO_4_ that is either bound in a dilute form within the EPS or forming colloidal matter on which the biofilm can develop. Both iron and sulfate concentrations used in this study are in the range of concentrations observed in the environment and in contaminated sites [50–52]. In order to increase the contrast and visualize the biofilm, we image the biofilm on a laboratory-based X-ray source using a relatively large source-to-detector (STD) distance. The large STD was used to exploit X-ray free space propagation in order to render visible refraction effects that take place as the X-rays travel through the sample, a technique commonly known as propagation-based phase contrast (PBI) [53, 54]. In addition, a robust Fourier filter which has the form of a Lorentzian function is applied as a digital pre-processing tool to the acquired projections to enhance the contrast and to improve the signal-to-noise ratio (SNR) [55, 56]. The presented method allows to image the 3D biofilm morphology in porous media at the mesoscale, using an X-ray lab source and a non-destructive contrast agent.

## Materials and methods

### Porous media and biofilm culturing

**Fig 1:**
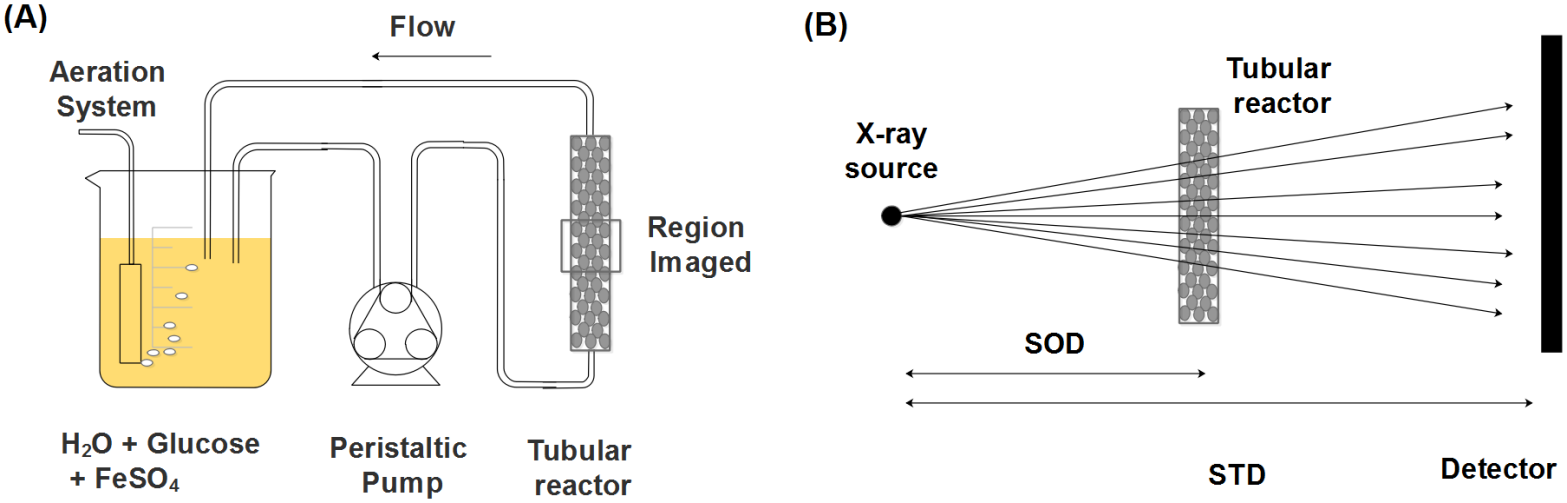
(A) Schematic of the experimental setup used for the biofilm culturing as well as the region of the tubular reactor used for biofilm imaging. (B) Schematic of the configuration used for the X-ray scans where the distances SOD and STD represent the source-to-object (SOD) and the source-to-detector distance (STD).

The porous media used in this study consisted of Nafion pellets (NR50 1100 EW, Ion Power, Munich, Germany) of 2.5 mm diameter similar to coarse sand grains. This material has similar physical and chemical properties to sand grains (e.g. particle size distribution or ion exchange capacity) and its optical refractive index in the visible domain is similar to that of water [57, 58]. For the inoculation of the media, the Nafion pellets were immersed for 24 h in an aerated batch containing 500 mL of natural water sampled from a pond located on the ETH campus. After 24 h, a polymethyl methacrylate (PMMA) tubular reactor (inner diameter 10 mm, length 160 mm) was wet packed with the pellets and connected to the batch. A biofilm was cultivated for 7 days using the setup shown in Fig 1A. A 500 mL feed solution made of tap water containing 1 g/L of glucose and 100 mg/L was used and changed every 48 h. A volumetric flow rate of 5 mL/min was set which corresponds to a Darcy velocity *q* of 1.06 mm/s. The initial porosity (volumetric fraction of the liquid phase) *ϕ* is ca. 40%, which yields an average pore scale velocity *v*_*p*_ ≈ *q*/*ϕ* = 2.65 mm/s and corresponding Reynolds number *Re* = *qd*/*ν* ≈ 2.5 and Péclet number *Pe* = *qd*/*D*_*H*_2_*O*_ ≈ 1000. Upon biofilm growth, the porosity decreased by a twofold factor, meaning that the average pore scale velocity doubled.

### Contrast agents

A concentration of 100 mg/L of the contrast agent used in this study FeSO_4_x7(H_2_O) (corresponding to 56 mg/L FeSO_4_ (20.6 mg/L Fe and 35.4 mg/L SO_4_) or 0.37 mmol/L FeSO_4_) was continuously added to the feed solution during the biofilm growth. The concentrations of both iron and sulfate compounds are in the range of concentrations observed in the environment [50–52] or used for other experimental work [32, 45, 46]. At that concentration and for the initial pH observed in this study (pH ≈ 7), the iron should be still soluble. However, the oxidation to Fe(III), which is less soluble and tends to form colloids, should occur quite rapidly [52]. In fact, iron flocs formed a few hours after the start-up of the system. Therefore, the inlet tubing was carefully set close to the water level, to limit the circulation of the flocs and to enhance their settling. The biofilm grown here exhibited a brownish color typical of iron oxides. Before the X-ray measurements, a volume of water corresponding to 8 times the initial pore volume was injected through the flow cell to ensure that no biofilm unbound iron was remaining in the liquid phase.

To investigate the influence of the colloidal and flocculated iron on porous medium flow and to monitor deposition of colloids inside the medium, we performed a control experiment. In that experiment, a FeSO_4_ containing solution was recirculated through a packed tubular reactor with identical conditions as those stated above, but without inoculation.

In order to compare the results obtained from this new approach with an already existing method, we followed the approach presented by Davit et al. [39], using a particulate Micropaque^®^ (Guerbet, Zurich) BaSO_4_ suspension as a contrast agent that was introduced into the liquid phase after the growth of the biofilm. BaSO_4_ suspensions are used to image biofilms in porous media because of the high attenuation coefficient of barium. Another advantage is that the BaSO_4_ particles are micrometer-sized and should therefore be physically size excluded from the EPS matrix [44]. Micropaque^®^ suspensions have a particle size distribution close to 1 *μm* (according to the manufacturer of the product, 25% ≤ particles larger than 2 *μm*, 20% ≤ 0.5*μm* an average diameter of 1.25 *μm*). These suspensions also contain additional stabilizing agents (e.g. xanthan gum, polydimethylsiloxane (PDMS) or sodium citrate etc.) that prevent the aggregation and sedimentation of the particles but strongly influence the rheological properties of the suspension. Plouraboué et al. [59] showed that the Micropaque^®^ barium suflate suspensions exhibited a viscosity much higher than water and a shear-thinning behavior similar to that of blood (see Fig 1.b in Plouraboué et al. [59]). Here, a Micropaque^®^ suspension of 0.1 g/L BaSO_4_ concentration was injected in the tubular reactor at 10% of the volumetric flow rate applied during the biofilm culturing in an attempt to avoid forced detachment due to the injection of the contrast agent. Due to dispersion and dilution effects, the distribution of the BaSO_4_ in the reactor was inhomogeneous after 1 pore volume. Therefore, 2 times the initial pore volume of the tubular were injected to completely saturate the tubular reactor with the suspension. Although the injection was done carefully and at a flow rate 10 times smaller than the one used for water, significant biofilm detachment was visually observed during the injection.

### X-ray imaging

Table 1 provides an overview of the different imaging conditions and image analysis approaches for the four datasets obtained from three different scans performed in the frame of this work. A first scan was performed with the sample containing the biofilm stained with FeSO_4_ only. For this scan (used to obtain the FeSO_4_ and LFeSO_4_ datasets), the source-to-object distance (SOD) was of 130 mm and the source-to-detector distance (STD) was set to the largest possible distance, 2330 mm (see Fig 1B). As mentioned previously, this fairly large STD distance was chosen to enhance the refraction effects occuring as the X-rays travel through the sample. The custom made tomographic setup was equipped with a microfocus X-ray tube (Viscom XT9160-TDX) and a 40 x 40 *cm*^2^ flat panel detector (Perkin Elmer XRD 1621) with 200 x 200 *μm*^2^ pixels. Since biofilms weakly absorb X-rays, the source had to be operated at a voltage of 50 *kV* and and a focused electron beam current (FEC) of 190 *μA*. 1441 projections with a field of view (FOV) of 1.85 x 1.85 *cm*^2^ were gathered at 0.25° angle steps with two frames per projection at a resolution of 9 *μm*. For the second scan (BaSO_4_ dataset), the same sample was used (see Table 1). The SOD distance was unchanged and the STD distance was reduced to 1017 mm, which would correspond to a reasonable distance for standard X-ray attenuation-based imaging. The voltage was set to 80 *kV* and the FEC to 120 *μA*. 1441 projections with a FOV of 4.3 x 4.3 *cm*^2^ were gathered at 0.25° angle steps with three frames per projection at a resolution of 21 *μm*. The difference in resolution between both scans is due to the fact that the SOD stayed constant and the STD decreased, reducing the physical magnification. For the last scan (control dataset LControl), the settings were the same as for the first one. Due to the rather low contrast of the projections and variations in the source intensity the projections were normalised to reach a reasonable contrast in the 3D volume. This pre-processing and the reconstruction were performed on in-house developed software tools based on filtered back-projection [60]. The scanning times were of ca. 3 hours.

**Table 1:** Information relative to the different scans and datasets used in this work as well as the corresponding details concerning the data analysis.

Dataset	*FeSO*_4_	*LFeSO*_4_	*BaSO*_4_	*LControl*
Tubular reactor Nr	1	1	1	2
Scan Nr	1	1	2	3
Biofilm Growth	Yes	Yes	Yes	No
Contrast agents	*FeSO*_4_	*FeSO*_4_	*FeSO*_4_&*BaSO*_4_	*FeSO*_4_
Source to detector distance (mm)	2330	2330	1017	2330
Scan details	50 kV, 190 *μA*	50 kV, 190 *μA*	80 kV, 190 *μA*	50 kV, 190 *μA*
Scan resolution (*μm*)	9	9	21	9
Projection image processing prior to reconstruction	-	Lorentzian filter	-	Lorentzian filter
Image filtering prior to segmentation	-	3D curvature-driven diffusive filter	3D curvature-driven diffusive filter	3D curvature-driven diffusive filter
Segmentation approach	-	VG Max surface determination	Seeded region growing algorithm & VG Max surface determination	VG Max surface determination
Registered dataset	No	Yes	Yes	No

### Image analysis

#### Image preprocessing by means of a Lorentzian filter

A key step for the results produced with the FeSO_4_ data is the application of an image processing Lorentzian filter in Fourier space to each radiographic projection before tomographic reconstruction. To evaluate the utility of the Lorenztian filter, the data from the first scan was reconstructed with and without applying this filter (LFeSO_4_ resp. FeSO_4_ datasets) and also applied to the control dataset (LControl, see Table 1). The filter presented here substantially reduces noise and enhances contrast. It is based on the original work of [61] where it was initially developed as a method to retrieve phase-and-amplitude information from inline propagation-based X-ray holograms using monochromatic beams and specifically for single-material samples. Here, the monochromaticity and single material assumptions are discarded and the algorithm is used *strictly* as an image processing tool, which exploits its high numerical stability under the presence of noise. This stability was shown in previous studies to greatly improve the contrast and the signal quality both in simple projection imaging as well as tomography [55, 56, 62–66]. The Lorentzian filter used here has the following form:

$$\begin{array}{c} {\text{I}}^{\text{Filt}}={\text{F}}^{-1}[\frac{1}{\alpha {\text{k}}_{⊥}^{2}+1}\text{F}\{{\text{I}}^{\text{Rad}}\}] \end{array}$$ (1)

**Fig 2:**
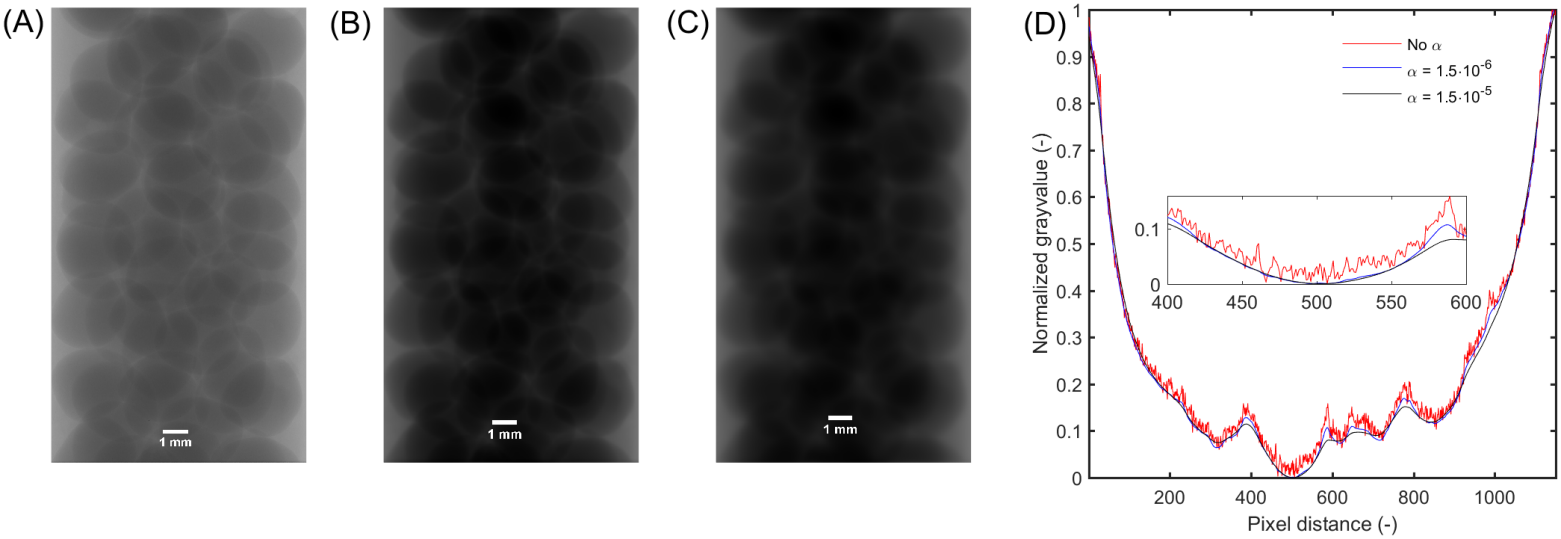
(A) raw projection image (unfiltered). (B) Lorentz filtered image of (A) using *α* = 1.5 ⋅ 10^−7^. (C) Lorentz filtered image of A using *α* = 1.5 ⋅ 10^−8^. The same dynamic range was used for (A), (B) and (C) for the sake of comparison. (D) displays horizontal normalized profiles at the location of the dashed line in (A), and (B) and (C) as well as for additional values of *α*. The inset shows a magnification of the gray value profile at the center of the tubular reactor. The scale bar in (A) is also valid on (B) and (C).

Here, I^Rad^ is the acquired radiographic image at a particular orientation. I^Filt^ is the filtered image once the Lorentzian filter is applied to I^Rad^. The symbols F and F^-1^ are the forward and inverse Fourier transforms. **k**_⊥_ = (*k*_*x*_, *k*_*y*_) are the transverse Fourier space coordinates reciprocal to the real space coordinates **r**_⊥_ = (*x*, *y*). In other words, the operation in Eq (1) is as follows: (i) Take the Fourier transform of the image I^Rad^, which in this case serves as input data; (ii) multiply the result by the Fourier space Lorentzian function $\frac{1}{\alpha {\text{k}}_{⊥}^{2}+1}$ and; (iii) take the inverse Fourier transform thus attaining the filtered image I^Filt^; (iv) perform the tomographic reconstruction using the backprojection method mentioned earlier. The filter was implemented as a Matlab^®^ routine [67]. The non-negative real valued constant *α* is an important parameter of the filter. It is important to mention that in the original form of the algorithm (see [61]), the value of *α* was known *a priori* since it is meant specifically to deal with only single material specimens. However, for our purposes, we use it as a *tuning parameter* that may be adjusted according to how much noise one desires to remove without over-blurring key features (edges) in the reconstructions. To obtain a good estimate of *α* we use a strategy that takes a raw projection image from the CT set which is used as reference and then graphically compared to a filtered image. An example of this strategy is seen in Fig 2. Here, we have an unfiltered radiographic image I^Rad^ in Fig 2A and a Lorentz filtered I^Filt^ version of the same image in Fig 2B and 2C obtained with values of *α* of 1.5 ⋅ 10^−6^ and with 1.5 ⋅ 10^−5^, respectively. In this work, an *α* value of 1.5 ⋅ 10^−6^ was used. In Fig 2D, gray value profiles of images (A), (B) and (C) across the same horizontal line are shown enabling a graphical comparison. These profiles show how the blue curve corresponding to (B) displays significantly less noise than the red curve corresponding to (A). In addition to noise reduction we see that the blue profile corresponding to (B) also follows the same trend than the red one. This is illustrated more clearly in the inset showing a magnified region of the profiles. For the other value of *α* (*α* = 1.5 ⋅ 10^−5^), both (C) and (D) (black), the effect of the over-blurring is evident. The balance of noise reduction and trend matching provides us a qualitative, yet clear visual indication whether our value chose for *α* is neither over- nor underestimated.

**Fig 3:**
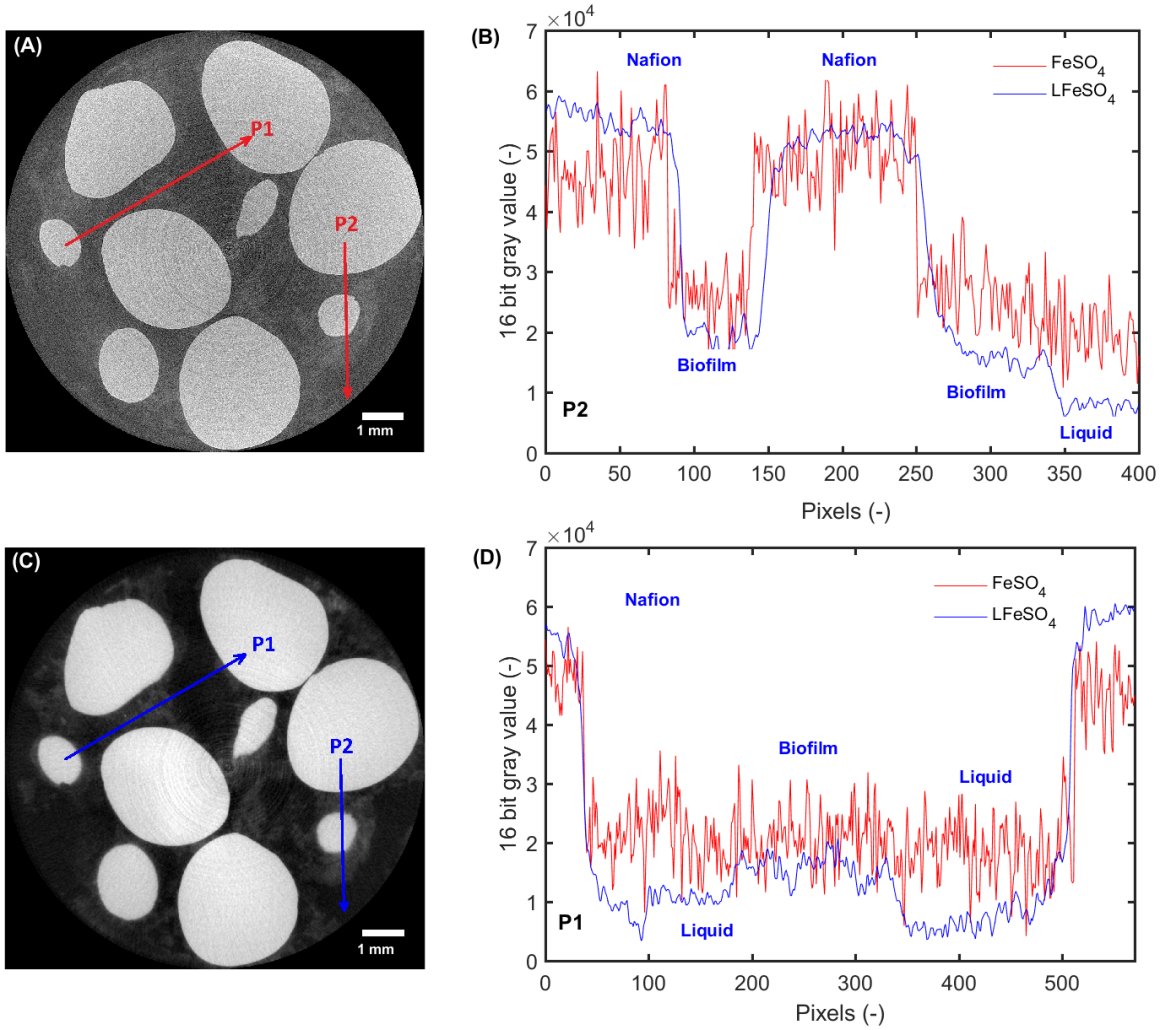
Slices from the FeSO_4_ (A) and LFeSO_4_ (C) datasets. For the sake of comparison, both images were normalized with 0.4% of the pixels saturated. The two red resp. blue arrows indicate the location and direction at which the gray value profiles are extracted. The scale bar represents 1 mm. Gray value profile for the first (P1, (D) and the second location P2, (B)). The profiles are labeled with the different phases observed.

The effectiveness of the Lorentzian filter from a tomographic perspective is illustrated in Fig 3. Here, we show tomographic slices of the same region with (A) and without (C) applying the Lorentzian filter before reconstructing. From this example it is evident that applying the Lorentzian filter prior to reconstruction greatly improves the visualization of the biofilm/water interface in the tomographic reconstruction. Fig 3B and 3D show gray value profiles labeled P1 and P2 clearly illustrating a reduction of noise. Boundaries still remain well defined after the application of the Lorentzian filter.

#### Segmentation

**Fig 4:**
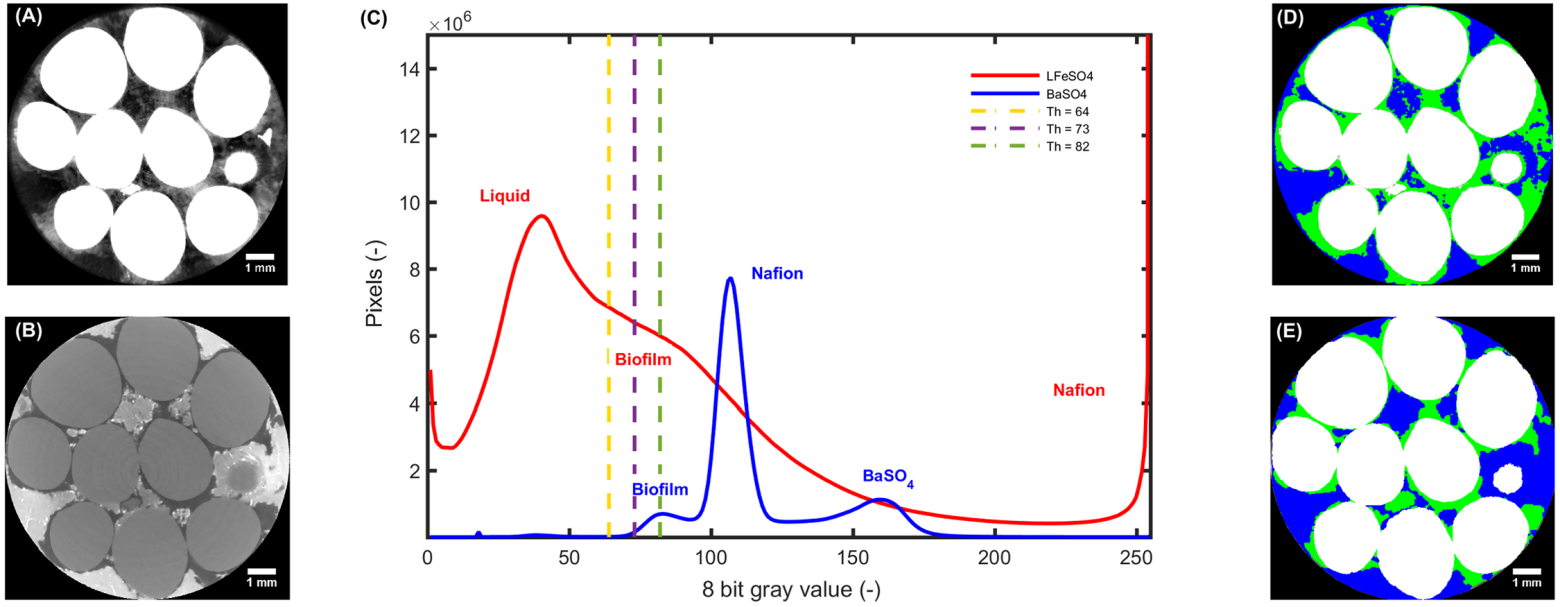
Middle slices (filtered prior to segmentation according information in Table 1) for the *LFeSO*_4_ (A) and *BaSO*_4_ (B) datasets. The corresponding 8 bit gray value histograms are shown in C) for the *BaSO*_4_ (blue) dataset and for the *LFeSO*_4_ (red) dataset after contrast enhancement and application of the 3D curvature-driven diffusive filter. For the *LFeSO*_4_ dataset, the vertical dashed lines in yellow, purple and green correspond to isosurface values of 64, 73 and 82 used for the segmentation and the corresponding sensitivity analysis. The peaks corresponding to the different phases are annotated. (D) and (E) show the segmented datasets where the solid, liquid and biofilm phases are color coded in white, blue and green respectively. The scale bar represents 1 mm.

Since two different contrast agents were used, it was not possible to define one segmentation procedure and apply it to all datasets so that a single approach was defined for each contrast agent. After reconstruction of the LFeSO_4_ dataset, the image contrast was enhanced so that the Nafion grains were mostly saturated. A curvature-driven diffusion filter was then run in Avizo^®^ (5 iterations and standard parameters: sharpness = 0.9, anisotropy = 0.6), reducing the noise while preserving the edges. Fig 4A shows a slice (located at the middle of the stack) obtained after the filtering and Fig 4C (red) shows the 8 bit gray value histogram obtained for the whole stack. On that histogram, the peaks corresponding to the liquid and Nafion phases are clearly identifiable. The liquid phase peak exhibits a strong shoulder on its right side, that corresponds to the biofilm phase. The overlap between the liquid and biofilm regions as well as the strong tail exhibited by the biofilm shoulder are due to the heterogeneous gray value distribution of the biofilm region (see Fig 4A). In order to segment these two regions, we assumed two overlapping peaks for the biofilm and the liquid phase and fixed a threshold at the inflection point located between the liquid phase peak and the biofilm shoulder. The determination of this inflection point is related to some uncertainty (see Supplementary Information). We therefore performed a simple sensitivity analysis by defining three different thresholds at the center and lower resp. higher end of the shoulder region. These thresholds are illustrated in purple, resp. yellow and green in Fig 4C). The gray values used for the sensitivity analysis correspond to ca. 10% of the central gray value (purple). For all threshold values, a thresholding was performed in VG Max^®^. Then, a closing operation was done in order to fill in small voids that are considered as noise from each phase. A region growing algorithm was used to identify disconnected segments of biofilm or liquid (e.g. pores in the biofilm or floating biofilm bits) that were merged into the surrounding phase. Since we observed the formation and displacement of air bubbles during the flushing out of the remaining iron or the injection of BaSO_4_, the air bubbles were segmented and merged into the liquid phase. The result of this segmentation is shown in Fig 4D).

Fig 4B shows a slice (same location than for Fig 4A, in the middle of the stack) of the BaSO_4_ dataset obtained after application of the same 3D curvature driven filter. The corresponding histogram of the BaSO_4_ dataset (see Fig 4C, in blue) shows a peak for the Nafion grains (central peak) and two additional peaks for the biofilm and the liquid phase. As it it shown in Fig 4B, due to the high attenuation of BaSO_4_, this dataset exhibited some beam hardening artifacts. These artifacts were overcome in the segmentation by using the ImageJ implementation [68, 69] of a seeded region growing algorithm [70] providing satisfying segmentation of the solid phase (see Supplementary Information for details). The liquid and biofilm phases were then obtained by thresholding after using the segmented solid phase as a mask. Fig 4E shows the corresponding segmented result. A sensitivity analysis was performed for that thresholding and is presented in the Supplementary Information. Finally, the control sample was imaged and segmented following the same approach as for the *LFeSO*_4_ dataset.

#### Registration of the two different datasets

A registration (volumetric image alignment onto a single coordinate system) of the two different tomograms was performed in order to compare locally (e.g. conditional probabilities) the volumetric fractions obtained from the different datasets. To begin with, the resolution of both datasets was matched. Then, a coordinate alignment process was performed in Matlab^®^ on the solid phases, because these are the most similar phases in both datasets. Here, the *imregister* function with default parameters was used for finding and optimizing an affine geometric transformation minimizing a mean square error metric. The optimization of the registration was performed using a regular step gradient descent method. All the results and analyses presented in the following were obtained from the registered datasets.

## Results

**Fig 5:**
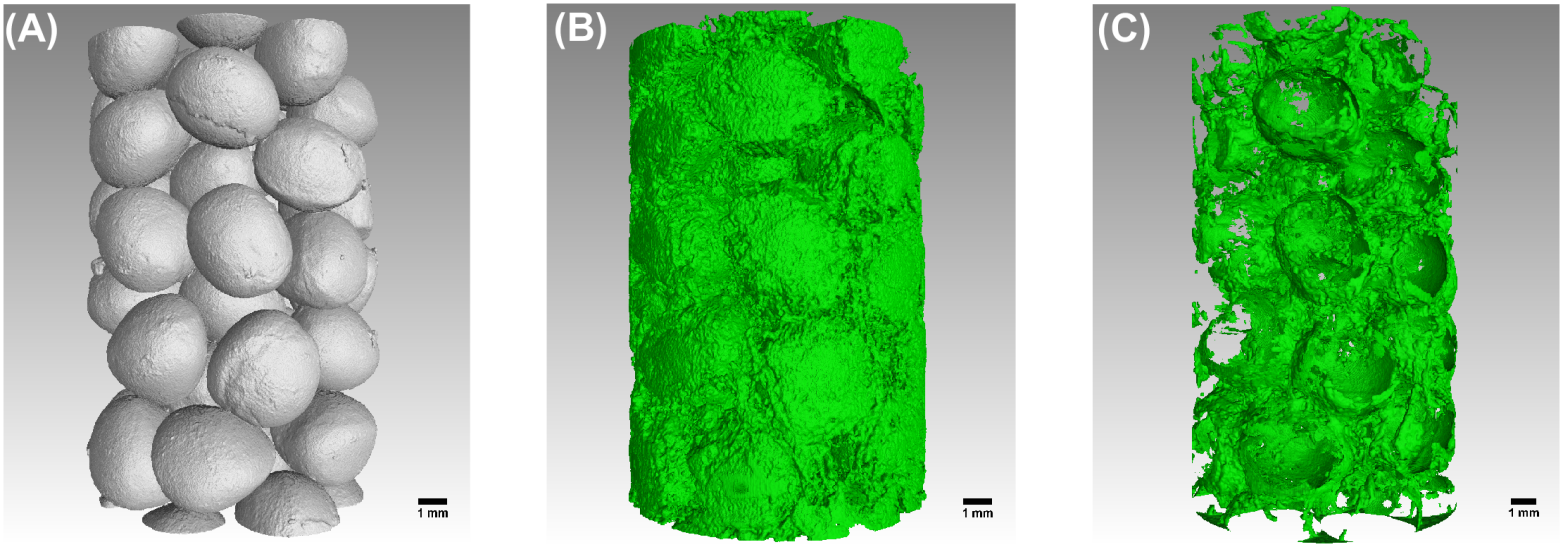
Three-dimensional renderings of the solid phase (left), of the sample imaged with FeSO_4_ (center) and barium suflate (right) as a contrast-enhancing agents.

Fig 4(A) and 4(B) show slices of the LFeSO_4_ and BaSO_4_ datasets obtained after filtering with a 3D curvature-driven filter. On both images, the biofilm is visible (light gray zones in) A) and the darkest ones in (B) but it seems that there is substantially more biofilm on image (A). The biofilm gray values in (A) exhibit an important heterogeneity. On this image, it is possible to identify darker zones belonging to the liquid phase and lighter ones to the biofilm. Fig 4(D) and 4(E) show the corresponding segmented slices where the segmented biofilm phases obtained with both contrast agents roughly overlap. However, on both the pre-processed (Lorentzian when used and 3D curvature-driven filtering) and segmented images, some biofilm regions locally do not match. Fig 5. shows 3D renderings of the solid phase (Nafion grains) and the segmented datasets which reveal that the volume fraction of the biofilm (green) is much smaller for the BaSO_4_ dataset. Qualitatively, the biofilm appears more patchy for the BaSO_4_ than for the LFeSO_4_ dataset. For the latter, the biofilm exhibits complex corrugated shapes that are more interconnected. It appears that greater detail of the biofilm morphology is resolved when LFeSO_4_ is used as a contrast enhancing agent.

**Fig 6:**
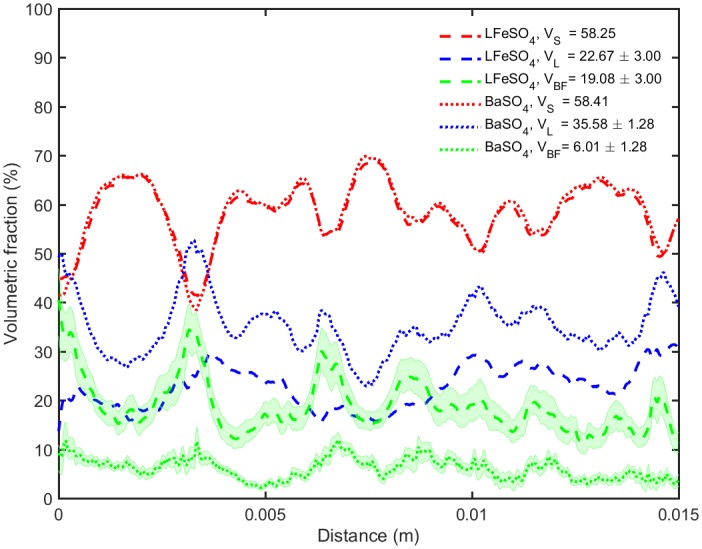
Profiles of the volumetric fractions (S: solid, L: liquid, BF: biofilm) obtained for the different datasets (BaSO_4_: small dashes, FeSO_4_: longer dashes). The shaded region is defined by the results obtained for the threshold sensitivity analysis. For the sake of clarity, the results of this sensitivity analysis are not added to the liquid phases. The average volumetric fractions (in percent) for the different phases (Solid *V*_*S*_, Liquid *V*_*L*_, Biofilm *V*_*BF*_) obtained with the two different contrast-enhancing agents is given in the legend.

Fig 6. shows volumetric fraction profiles along the streamwise direction for the LFeSO_4_ and for the BaSO_4_ datasets. The average total volumetric fractions obtained are given in the legend. The profiles obtained for the solid phase of both samples show a very good overlap and have volumetric fractions that are relatively close, at about 60% of the whole volume of the sample. The oscillations of these values present a wave length of ca. 2.5 mm approximately corresponding to the grain size diameter, indicating that there is some degree of order in the packing. While there is a good matching for the volumetric fractions profiles obtained for both solid phases, it is not the case for the remaining phases. For instance, the biofilm fraction obtained for the LFeSO_4_ dataset is about three times larger than for the BaSO_4_ dataset. The shaded region is delimited by the lower and higher thresholds used for the sensitivity analysis. Over the whole column, the biofilm volumetric fraction obtained with FeSO_4_ is consistently larger than for the BaSO_4_ dataset (about twice as much).

To study how the phases overlap in 3D space, we locally computed the conditional probabilities of the phase overlap between the two different datasets, e.g. the percentage of a first phase corresponding to the same phase in a second one (*P*(*A* ∣ *B*) = *P*(*B* ∩ *A*)/*P*(*B*)) (see Table 2). With more than 90% in both cases, the overlap is very good for the solid phases. Again, the remaining phases exhibit substantial differences. For the biofilm phase, the intersection of voxels identified as biofilm in both BaSO_4_ and LFeSO_4_ datasets only correspond to ca. 25% against ca. 70% for the opposite case. This indicates two different aspects: first that locally the volumetric fraction of the biofilm obtained for the LFeSO_4_ dataset is substantially higher than for the BaSO_4_ dataset. Secondly, as biofilm patches detached upon the BaSO_4_ injection, they might have reattached or filtered at some other locations within the column where there is no biofilm visible in the LFeSO_4_ dataset.

**Table 2:** Conditioned probabilities that a given phase in the FeSO4 data locally belongs to the same phase in the BaSO4 data computed for the solid (S), liquid (L) and biofilm (BF) phases for the registered Lorentz filtered FeSO_4_ and BaSO_4_ datasets.

Conditional probability	Percentage
*P*(*S*_*BaSO*_4__ ∣ *S*_*LFeSO*_4__)	93.85
*P*(*S*_*LFeSO*_4__ ∣ *S*_*BaSO*_4__)	98.18
*P*(*L*_*BaSO*_4__ ∣ *L*_*LFeSO*_4__)	88.20
*P*(*L*_*LFeSO*_4__ ∣ *L*_*BaSO*_4__)	54.25
*P*(*BF*_*BaSO*_4__ ∣ *BF*_*LFeSO*_4__)	24.74
*P*(*BF*_*LFeSO*_4__ ∣ *BF*_*BaSO*_4__)	72.57

The control sample was treated in the same way than the FeSO_4_ data set, except for the fact that it was not inoculated with the bacterial inoculum and that no growth medium was added for the 7 days. The image processing (filters, reconstruction and segmentation) used was also the same as the one used for the FeSO_4_ data set. The aim of this control was to estimate the concentration of colloidal FeSO_4_ in the biofilm itself. For the control, a colloidal volumetric fraction of about 10% was obtained, indicating that the iron equalled ca. 54% of the volumetric fraction of the biofilm. This indicates that the inorganic content of the biofilm cultivated here is fairly high.

## Discussion

In this study, the combination of a non-destructive contrast agent, a long STD and Lorentzian filtering revealed intact 3D biofilm morphologies in porous media. This particular combination of contrast agent and imaging tools borrowed from phase contrast imaging allowed to substantially reduce noise and improve the contrast of the sample containing materials with small attenuation coefficient differences.

**Fig 7:**
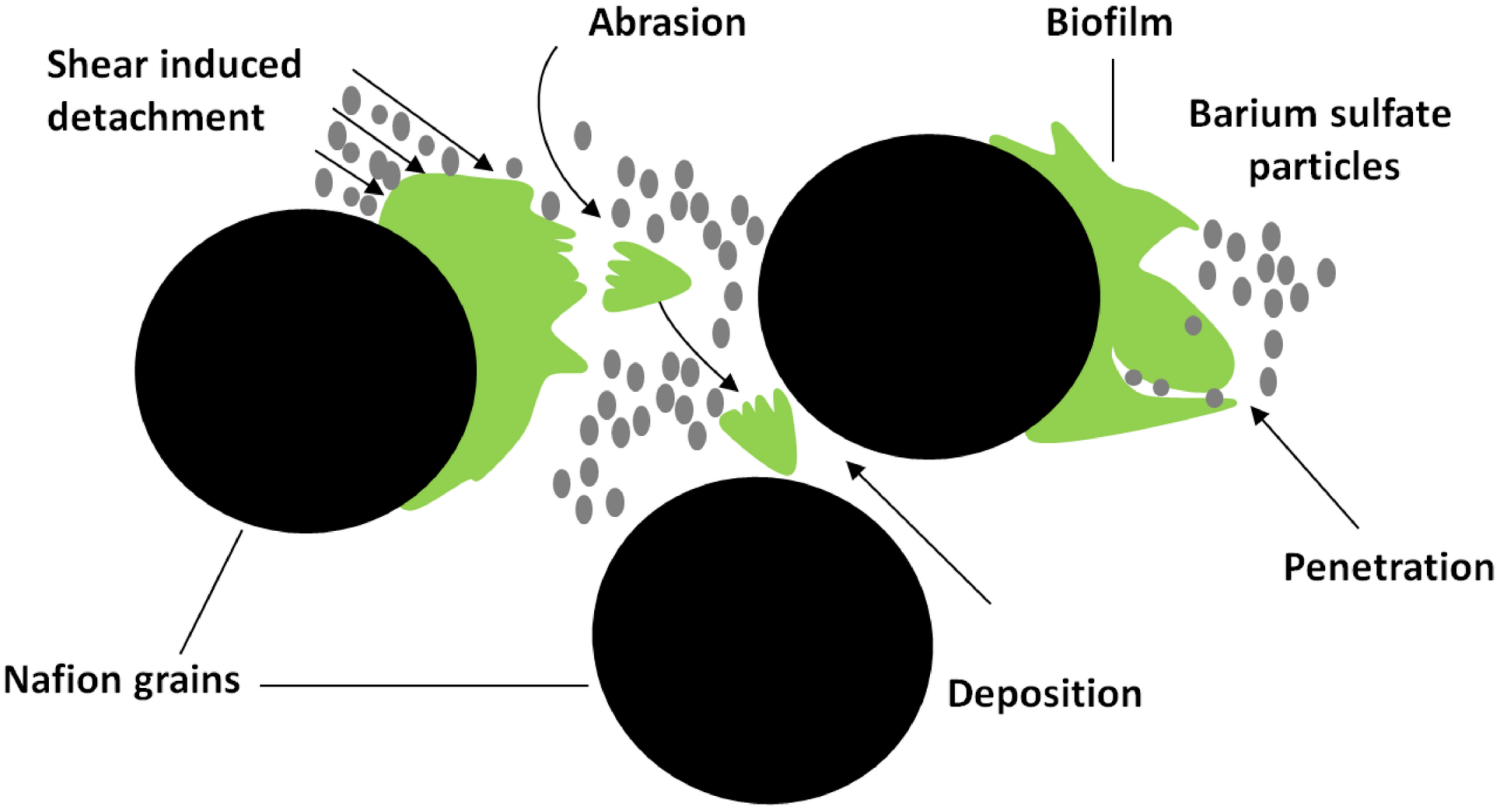
Schematic of biofilm detachment mechanisms during BaSO_4_ injection.

The different volumetric fraction obtained for the BaSO_4_ and FeSO_4_ contrast agents could be explained by the following reasons: uncertainty related to the segmentation or the registration, partial volume effects due to the different imaging resolution and interactions between the BaSO_4_ suspension and the biofilm (e.g. penetration or invasion of the biofilm and biofilm detachment upon the introduction of the contrast agent). The volumetric fraction obtained for the biofilm is sensitive to the threshold considered, but in all cases stays consistently higher than the BaSO_4_ volumetric fraction. The good agreement of both the average volumetric fractions and volumetric fractions profiles of the solid phase illustrated in Fig 6. allows us to rule out the uncertainty related to the registration or the partial volume effects as a main cause for the mentioned substantial differences. Therefore, penetration of the smallest BaSO_4_ particles within the biofilm and biofilm detachment (as illustrated schematically in Fig 7.) appear to be at the root of the differences observed.

About 20% of the BaSO_4_ particles used have a diameter smaller than 0.5 *μm* and are smaller than the largest biofilm pores expected [44]. In the present case, it is realistic to assume that a portion of the smallest BaSO_4_ particles could enter the biofilm channels (see Fig 7.) and therefore labelling the parts of the biofilm as liquid.

As mentioned earlier, biofilm detachment was observed during the injection of BaSO_4_ (see Supplementary Information). This observation was already noted previously in another study [39]. We estimated the average wall shear stress exerted by the BaSO_4_ suspension on the biofilm assuming a power law fluid flowing in a simplistic representation of an average pore in our system. Although the flow rate was 10 times smaller during the BaSO_4_ injection than the growth flow rate, we found ca. 3 times higher wall shear stresses in the BaSO4 injection stage due to the high viscosity and shear thinning properties of the BaSO4 suspension. Given the extremely wide range of shear rates taking place in the actual porous medium [58], locally the wall shear stresses ratio might be significantly higher, causing the detachment observed. In such a case, the rheological properties of the BaSO_4_ suspension should not be neglected.

Derlon et al. [71] and Rochex et al. [72] showed that biofilm form stratified structures and that the biofilm resistance to shear or shear strength [73] is dependent on the wall-normal location within the biofilm. Möhle et al. [46] showed that by increasing the biofilm shear strength, high concentrations of iron in biofilms had a positive effect on the biofilm stability. Here, lower shear strength regions of the biofilm probably detached upon BaSO_4_ injection and the basal layers with higher shear strength remain attached. Abrasion of biofilm by particles is another known detachment mechanism [71]. It is not excluded that BaSO_4_ particles-biofilm interactions also contributed to the detachment observed. Fig 7. illustrates the different mechanisms potentially causing the detachment. The shear and abrasion induced detachment explain the differences in the overall and local biofilm volumetric fractions observed. In Table 2, the low probability of the biofilm phase obtained with BaSO_4_ to also belong to the biofilm phase obtained with FeSO_4_ might also point to the deposition or filtration of some detached biofilm patches further upstream within the column.

Table 3 summarizes key points on which the method by [39] and the method presented here can be evaluated. The FeSO_4_-based method allows to image biofilms in porous media using a non-toxic and non-destructive contrast agent on a X-ray lab source with movable detector. The combination of long STD and Lorentzian filter allows to substantially increase the signal to noise ratio. As mentioned earlier, the colloidal fraction observed for the control dataset (LControl) is not negligible. Nevertheless, the detected biofilm fraction in the LFeSO4 experiment is much higher which confirms that the higher volumetric fraction observed in the latter case is due to biological activity, either due to the biofilm itself or to precipitation induced by bacterial activity, (e.g. iron oxidation, sulfide salts production, see [5] and [52]). The BaSO_4_-based method provides clear contrast between the different phases but interactions of the BaSO_4_ suspension with the biofilm induce substantial biofilm detachment. In both cases, some uncertainty remains related to the segmentation, which could however be quantified.

**Table 3:** Evaluation of the presented method and another existing one for imaging biofilms in porous media.

	*LFeSO*_4_	*BaSO*_4_
Biofilm integrity	non-destructive	can cause detachment during *BaSO*_4_ injection
Biofilm toxicity	none, but high inorganic content biofilm	none
Lab XCT requirements	movable detector	none
Imaging issues	High signal to noise ratio	Prone to beam hardening
Reconstruction requirements	Lorenztian filter	none
Segmentation	Thresholding	Combination of region growing and thresholding

## Conclusion

In this paper, we presented an innovative method to image biofilms in porous media combining iron sulfate as a contrast agent, a long STD and a Lorentzian filter. The non-toxic and non-destructive contrast agent was continuously added to the biofilm during the biofilm growth. The combination of using a large STD together with application of a Lorentzian Fourier filter allowed to exploit refraction effects. The reconstructed data showed a substantial reduction in noise and an increase in the contrast between materials exhibiting low attenuation coefficients differences, revealing the biofilm morphology. We found that in the porous medium and for the present growth conditions, the biofilm exhibits complex corrugated structures. We compared this method with an existing method using BaSO_4_ as as a contrast agent for the exact same sample and observed some differences in the biofilm morphology obtained due to interactions between the biofilm and the BaSO_4_. Namely, due to abrasion and shear detachment, more than 50% of the biofilm was washed out by the contrast agent emphasizing the need for non-destructive contrast agents for biofilm imaging in porous media. The method presented in this study delivers 3D biofilm morphologies in porous media non-destructively on a X-ray lab source. Possible applications are studies addressing the interplay between biofilms, their morphology and local hydrodynamic and mass transport processes in realistic porous media models.

## Supporting information

S1 File Segmentation of the LFeSO_4_ data set. (PDF)

S2 File Segmentation of the BaSO_4_ data set. (PDF)

S3 File Effect of the rheological properties of the BaSO_4_ on the wall shear stress. (PDF)

S4 File Lorentzian Filer: Theoretical Background. (PDF)
