# Correction: Biofilm imaging in porous media by laboratory X-Ray tomography: Combining a non-destructive contrast agent with propagation-based phase-contrast imaging tools

**DOI:** 10.1371/journal.pone.0192099

**Published:** 2018-01-25

**Authors:** Maxence Carrel, Mario A. Beltran, Verónica L. Morales, Nicolas Derlon, Eberhard Morgenroth, Rolf Kaufmann, Markus Holzner

There is an error in the seventh sentence of the third paragraph beneath the “Contrast Agents” head of the Materials and Methods section. The correct sentence is: Here, a Micropaque^®^ suspension of 0.1 g/mL BaSO_4_ concentration was injected in the tubular reactor at 10% of the volumetric flow rate applied during the biofilm culturing in an attempt to avoid forced detachment due to the injection of the contrast agent.
