# Supplementary material for: Biofilm imaging in porous media by laboratory X-Ray tomography: Combining a non-destructive contrast agent with propagation-based phase-contrast imaging tools
Source: PLoS One. 2017 Jul 21;12(7):e0180374. doi: 10.1371/journal.pone.0180374 (PMC5521744; doi:10.1371/journal.pone.0180374)
Supplement: S1 File — (PDF) [file pone.0180374.s001.pdf]

Biofilm Imaging in Porous Media by X-ray Tomography: Combining a Non-Destructive Contrast Agent with Propagation-Based Phase-Contrast Imaging Tools.

Maxence Carrel[1], Mario A. Beltran[2], Verónica L. Morales[1,3], Nicolas Derlon[1,4], Eberhard Morgenroth[1,4], Rolf Kaufmann[2], Markus Holzner[1*]

1 Institute of Environmental Engineering, ETH Zürich, Stefano Franscini-Platz 5, 8093 Zurich, Switzerland
2 Swiss Federal Laboratories for Materials Science and Technology (EMPA), Dübendorf, Switzerland
3 Department of Civil and Environmental Engineering, University of California, Davis, California, USA
4 Swiss Federal Institute of Aquatic Science and Technology (EAWAG), Dübendorf, Switzerland

* holzner@ifu.baug.ethz.ch

S1 File. Segmentation of the $LFeSO_4$ data set

Fig.1 shows an extract of the histogram of the $LFeSO_4$ data set after the Lorentzian filtering and the pre-processing step (contrast enhancement and 3D curvature-driven diffusive filter). The second derivative of the histogram is taken to determine the gray value that separates the biofilm phase from the liquid phase (see Fig. 1, green curve). The facts that the second derivative intersects several times the horizontal axis shows that there is some uncertainty related to the inflection point determination. The yellow, purple and green dashed vertical lines represent the three different 8 bit gray value thresholds of 64, 73 and 82 chosen in order to take this uncertainty into account. These three different threhsolds allow to assess the uncertainty of the inflection point determination on the final segmentation. Fig.2 shows contours of the interface between the liquid and biofilm at the three selected gray values on a vertical cross section of the sample. Fig.2A) is used as reference for an unobstructed view of the cross section. Fig. 2 B-D shows the contour at gray value thresholds of 64, 73 and 82, respectively.

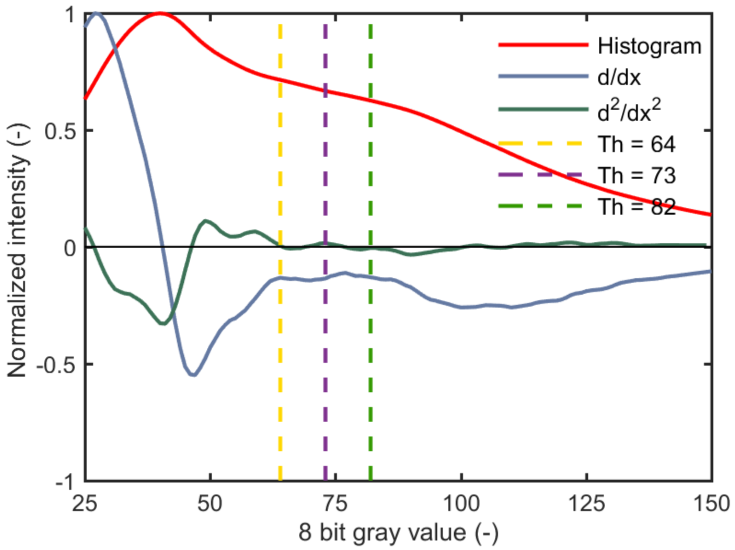

Fig 1. Extract of the gray value histogram for the $LFeSO_4$ data set (red). The first and second derivative of the histogram are shown in gray respectively dark green. The yellow, purple and light green dashed vertical lines at 8 bit gray value of 64, 73 and 82 represent the uncertainty zone considering the inflection point determination.

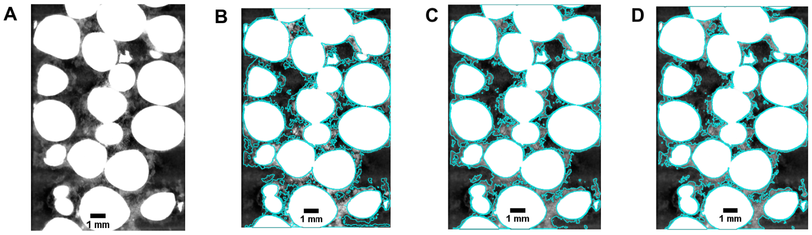

Fig 2. A) raw image of the $FeSO_4$ data set. B), C) and D) : biofilm phases obtained for the $FeSO_4$ data set based on 8 bit gray value thresholds of 64, resp. 73 and 82.
