# Supplementary material for: Biofilm imaging in porous media by laboratory X-Ray tomography: Combining a non-destructive contrast agent with propagation-based phase-contrast imaging tools
Source: PLoS One. 2017 Jul 21;12(7):e0180374. doi: 10.1371/journal.pone.0180374 (PMC5521744; doi:10.1371/journal.pone.0180374)
Supplement: S2 File — (PDF) [file pone.0180374.s002.pdf]

Biofilm Imaging in Porous Media by X-ray Tomography: Combining a Non-Destructive Contrast Agent with Propagation-Based Phase-Contrast Imaging Tools.

Maxence Carrel[1], Mario A. Beltran[2], Verónica L. Morales[1,3], Nicolas Derlon[1,4], Eberhard Morgenroth[1,4], Rolf Kaufmann[2], Markus Holzner[1*]

1 Institute of Environmental Engineering, ETH Zürich, Stefano Franscini-Platz 5, 8093 Zurich, Switzerland
2 Swiss Federal Laboratories for Materials Science and Technology (EMPA), Dübendorf, Switzerland
3 Department of Civil and Environmental Engineering, University of California, Davis, California, USA
4 Swiss Federal Institute of Aquatic Science and Technology (EAWAG), Dübendorf, Switzerland

* holzner@ifu.baug.ethz.ch

S2 File. Segmentation of the $BaSO_4$ data set

Fig.1 shows the histograms of the $BaSO_4$ data set (blue curve) and after substraction of the solid phase segmented with the seeded region growing algorithm (dashed blue). Due to the beam hardening artifacts caused by high attenuation of the $BaSO_4$ suspension, the gray value distribution corresponding to $BaSO_4$ occupied pores (i.e., liquid) was strongy tailed. A threshold was visually defined for a value of 110 and a sensitivity analysis on that threshold value was performed. Similar to Fig. 2 int the File S1, Fig.2 shows a cross section of the sample, with contours of the interface between the liquid and biofilm at three threshold gray values. Variation of the thresholded yielded in overall volumetric fraction variations of 1.28%. Thresholds outside this interval have also been tried but lead to inconsistent delineations of the biofilm-liquid boundary.

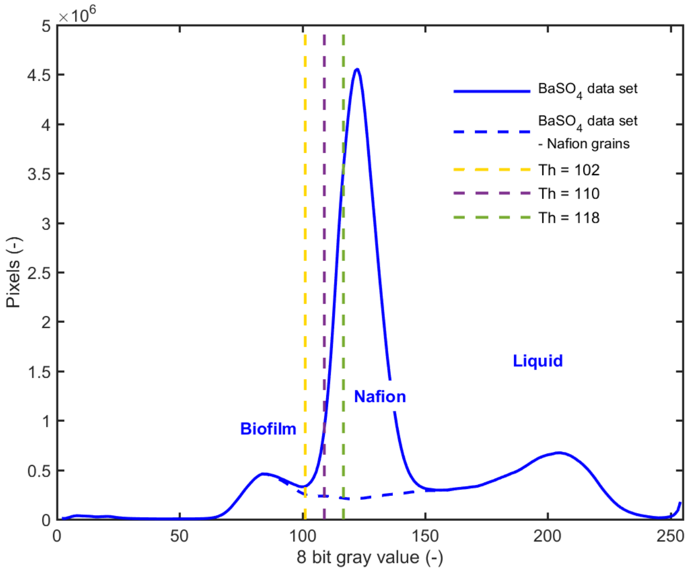

Fig 1. Histogram of the whole $BaSO_4$ data set (blue) and after the substraction of the solid phase obtained with the seeded region growin algorithm (dashed blue). The yellow, purple and green vertical lines represent the 8 bit gray value threhsolds of 102, 110 and 118 used for the sensitivity analysis.

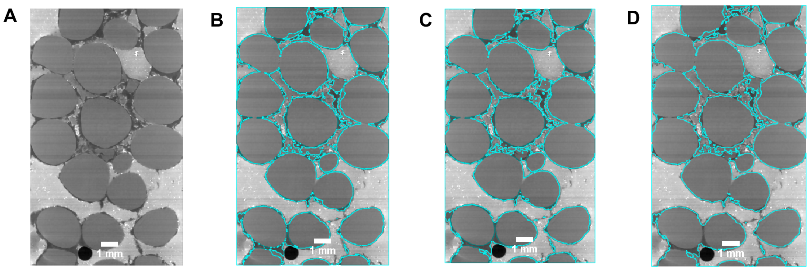

Fig 2. A) raw image of the $BaSO_4$ data set. B), C) and D) : liquid phases obtained for the $BaSO_4$ data set based on 8 bit gray value thresholds of 102, resp. 110 and 118.
