# Supplementary material for: Biofilm imaging in porous media by laboratory X-Ray tomography: Combining a non-destructive contrast agent with propagation-based phase-contrast imaging tools
Source: PLoS One. 2017 Jul 21;12(7):e0180374. doi: 10.1371/journal.pone.0180374 (PMC5521744; doi:10.1371/journal.pone.0180374)
Supplement: S3 File — (PDF) [file pone.0180374.s003.pdf]

# Biofilm Imaging in Porous Media by X-ray Tomography: Combining a Non-Destructive Contrast Agent with Propagation-Based Phase-Contrast Imaging Tools.

Maxence Carrel[1], Mario A. Beltran[2], Verónica L. Morales[1,3], Nicolas Derlon[1,4], Eberhard Morgenroth[1,4], Rolf Kaufmann[2], Markus Holzner[1*]

**1** Institute of Environmental Engineering, ETH Zürich, Stefano Franscini-Platz 5, 8093 Zurich, Switzerland
**2** Swiss Federal Laboratories for Materials Science and Technology (EMPA), Dübendorf, Switzerland
**3** Department of Civil and Environmental Engineering, University of California, Davis, California, USA
**4** Swiss Federal Institute of Aquatic Science and Technology (EAWAG), Dübendorf, Switzerland

* holzner@ifu.baug.ethz.ch

## S3 File. Effect of the rheological properties of the $BaSO_4$ on the wall shear stress

Here, we evaluate the influence of the rheological properties of the $BaSO_4$ suspension on the wall shear stress induced on the biofilm during the injection of the contrast agent. In order to do so, we consider a pore of diameter $D$ of 2.5 $mm$ representative of the average pore size in the tubular reactor used in this study. We set the volumetric flow rate, so that the Reynolds number obtained in this system is of 2.5 as for the tubular reactor. We assume steady laminar flow and use cylindrical coordinates $(R, \theta, z)$. In that case, the wall shear stress $\tau_0$ is a function of the pressure gradient $dp/dz$ and of the pore's diameter $D$ [1]:

$$\tau_0 = \frac{1}{4}\left|\frac{dp}{dz}\right| D \tag{1}$$

In this system, for the flow of the growth medium, the velocity profile is a parabolic function of the radius [1]:

$$v(r) = \frac{1}{16\eta_{H_2O}}\left|\frac{dp}{dz}\right|(D^2 - r2) \tag{2}$$

where $R$ is the radius of the cylinder and we assume the growth medium to have the same dynamic viscosity than water. The volumetric flow rate is obtained by integration [1]:

$$Q = \frac{\pi}{128}\left|\frac{dp}{dz}\right|\frac{D^4}{\eta_{H_2O}} \tag{3}$$

[2] showed that the $BaSO_4$ suspension exhibit a shear-thinning. We model the rheology of the suspension as a power law fluid with::

$$\eta_{BaSO_4}(\dot{\gamma}) = K\dot{\gamma}^{n-1} \tag{4}$$

where the dynamic viscosity $\eta$ is a function of the shear rate $\dot{\gamma}$. We use the data of [2] to approximate the flow consistency index $K = 0.048$ and the power law index $n = 0.6592$.

The velocity profile in pore is obtained combining the equation of motion in cylindrical coordinates and the definition of the dynamic viscosity made in Eq.4 (for details, see [1]),:

$$v(r) = \frac{nD}{2(1+n)} \left( \left| \frac{dp}{dz} \right| \frac{D}{4K} \right)^{1/n} \left[ 1 - \left( \frac{2r}{D} \right)^{1/n+1} \right] \tag{5}$$

For the $BaSO_4$ suspension, the flow rate is obtained after integration yielding:

$$Q = \frac{\pi D^3}{8} \frac{n}{1+3n} \left( \left| \frac{dp}{dz} \right| \frac{D}{4K} \right)^{1/n} \tag{6}$$

In the experiments, the volumetric flow rate used for the injection of the $BaSO_4$ was 10 times smaller than during the biofilm culturing. For the pore considered here, the resulting pressure gradient is 2.7 times larger for the $BaSO_4$ suspension than for the growth medium. As the wall shear stress scales linearly with the pressure gradient (see Eq.1), the increase in pressure gradient results in an increase in shear stress by nearly a factor of 3 in the presence of BaSO4. The corresponding streamwise velocity profiles are shown in Fig.1. The gradients exhibited by the $BaSO_4$ velocity profiles are substantially smaller than for the growth medium ($H_2O$). It has to be considered that for the shear rates observed, the dynamic viscosity obtained using the power law fluid model is around 80 times higher than for water, explaining the high wall shear stresses obtained despite of the small velocity gradients. This increase of wall shear stress could explain the detachment observed.

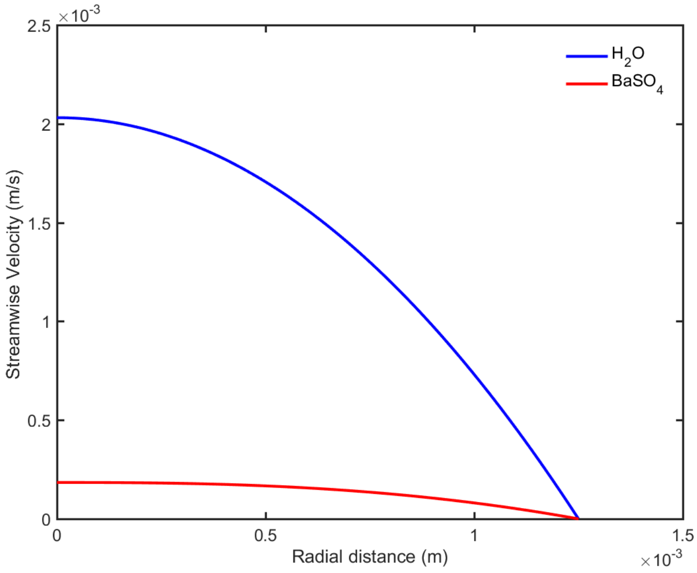


**Fig 1.** Velocity profile obtained for a flow in a representative pore for the growth solution and for the $BaSO_4$ suspension.

Moreover, it has to be noted that pore-scale velocities in porous media have an extremely wide distribution [3] and can deviate substantially from the mean velocity and consequently, the ratio of mean shear stress values exerted on the biofilm by the $BaSO_4$ or the growth medium can be much higher than the factor of 3 obtained here. In

such a case, the rheological properties of the $BaSO_4$ should not be neglected as they could substantially contribute to the biofilm detachment.

## Biofilm detachment upon injection of the $BaSO_4$ suspension

Fig. 2 shows an image of the biofilm tubular reactor before the $BaSO_4$ injection A) and during the injection B) and C). The biofilm shows a reddish color typical of biofilms with high iron oxides content. B) and C) show the heterogeneous distribution of the $BaS_4$ concentration along the column shortly after starting the injection, as some pores are saturated and others not, due to mixing and dispersion. During the injection, we visually observed biofilm detachment and patches moving through the column. The black arrow in Fig. 2 shows a biofilm patch that at early times was free of BaSO4 but at later times it was not, which suggests sloughing or BaSO4 penetration into the biofilm between the acquisition of the images B) and C).

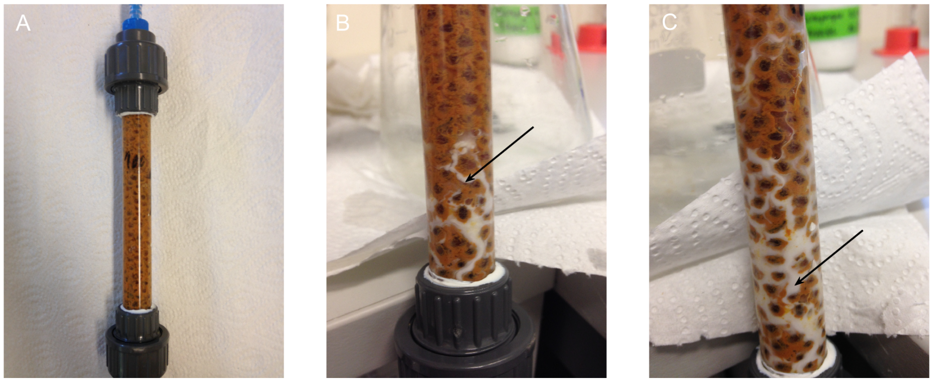


**Fig 2.** A) Biofilm tubular reactor used in this study. B) and C) images of the biofilm during the injection. The arrow is showing a biofilm patch beeing detached.

## References

1. Irgens F. In: Rheology and Non-Newtonian fluids. Springer International Publishing; 2014. p. 190.
2. Plouraboué F, Cloetens P, Fonta C, Steyer A, Lauwers F, Marc-Vergnes JP. X-ray high-resolution vascular network imaging. Journal of Microscopy. 2004;215:139–148.
3. Holzner M, Morales VL, Willmann M, Dentz M. Intermittent Lagrangian velocities and accelerations in three-dimensional porous medium flow. Physical Review E. 2015;92:013015.
