# Supplementary material for: Biofilm imaging in porous media by laboratory X-Ray tomography: Combining a non-destructive contrast agent with propagation-based phase-contrast imaging tools
Source: PLoS One. 2017 Jul 21;12(7):e0180374. doi: 10.1371/journal.pone.0180374 (PMC5521744; doi:10.1371/journal.pone.0180374)
Supplement: S4 File — (PDF) [file pone.0180374.s004.pdf]

Biofilm Imaging in Porous Media by X-ray Tomography: Combining a Non-Destructive Contrast Agent with Propagation-Based Phase-Contrast Imaging Tools.

Maxence Carrel[1], Mario A. Beltran[2], Verónica L. Morales[1,3], Nicolas Derlon[1,4], Eberhard Morgenroth[1,4], Rolf Kaufmann[2], Markus Holzner[1*]

1 Institute of Environmental Engineering, ETH Zürich, Stefano Franscini-Platz 5, 8093 Zurich, Switzerland
2 Swiss Federal Laboratories for Materials Science and Technology (EMPA), Dübendorf, Switzerland
3 Department of Civil and Environmental Engineering, University of California, Davis, California, USA
4 Swiss Federal Institute of Aquatic Science and Technology (EAWAG), Dübendorf, Switzerland

* holzner@ifu.baug.ethz.ch

S4 File. Lorentzian filter: Theoretical Background

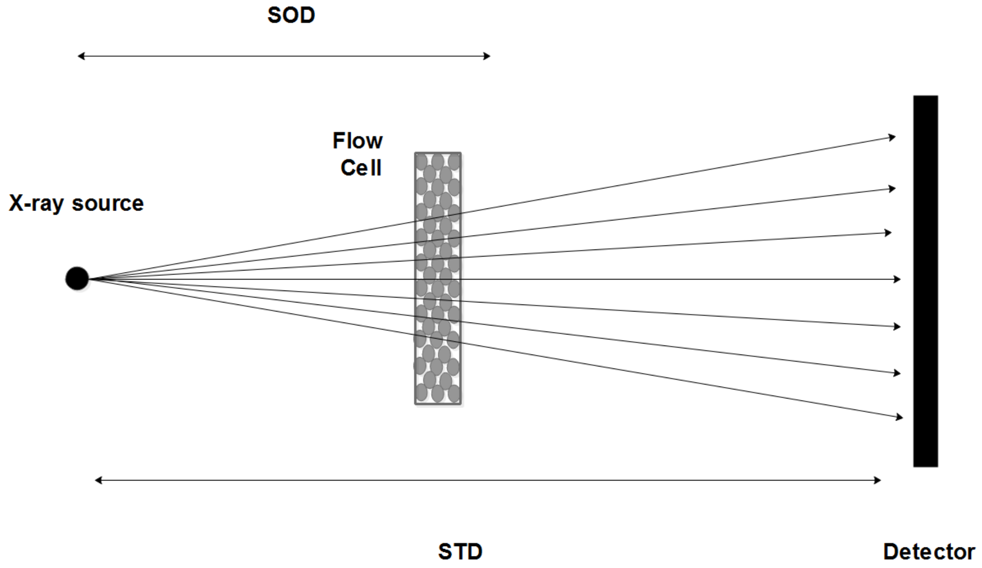

Fig 1. Schematic of the configuration used for the X-ray scans where the distances SOD , ODD and STD represent the source-to-object (SOD), object-to-detector (ODD) and the source-to-detector distance (STD), adapted from [1].

This appendix briefly reviews the theory phase retrieval (Paganin *et al.* [1] method) in the context of propagation-based phase contrast imaging (PBI). It was put to together with the aim of: (i) Assisting readers who are non-experts in the field of coherent X-ray imaging with a brief summary therefore avoiding extensive literature review; (ii) Re-derive Paganin *et al.* [1] single-image phase-retrieval method and demonstrate that one can arrive at the same form by including polychromatic effect from first principles and; (iii) Show how the method can adapted and utilized as an image processing tool as a "Lorentzian Fourier filter".

We begin by stating the Transport-of-Intensity equation (TIE) [2]. The TIE is often used as starting point for paraxially-propagating monochromatic beams, which we will later generalize by accounting for polychomaticity. It is a second order elliptic partial differential equation that describes the local conservation of optical energy as wavefields evolve from one plane $z = \mathrm{R}_1$ to another infinitesimally separated parallel plane $z = \mathrm{R}_2$. It has the structure of a continuity equation and can be viewed as such. The form is given by

$$\nabla_{\perp} \cdot [\mathrm{I}(\mathbf{r}_{\perp}, E, \mathrm{R}_1)\nabla_{\perp}\phi(\mathbf{r}_{\perp}, E, \mathrm{R}_1)] = -k\frac{\partial}{\partial z}\mathrm{I}(\mathbf{r}_{\perp}, E, z) \tag{1}$$

Here, $\mathrm{I}(\mathbf{r}_{\perp}, E, \mathrm{R}_1)$ (with the aid of Fig. 1 is the wavefield's intensity at the plane exiting the sample $z = \mathrm{R}_1$ and $\phi(\mathbf{r}_{\perp}, E, \mathrm{R}_1)$ is the wavefield's phase at that same plane. The cartesian coordinates $\mathbf{r}_{\perp} = (x, y)$ are used to describe the field plane transverse to the optic axis z. $E = hc/\lambda$ is the photon energy of the beam which has been kept arbirary for the moment. $k = 2\pi/\lambda$ is the wavenumber and the term $\frac{\partial}{\partial z}\mathrm{I}(\mathbf{r}_{\perp}, E, z)$ represents the first order derivative of the intensity along the propagation axis z (see Fig 1).

For normally incident plane-wave illumination of an optically thin homogeneous object, the intensity and phase at the contact plane $z = \mathrm{R}_1$ is given by:

$$\mathrm{I}(\mathbf{r}_{\perp}, E, \mathrm{R}_1) = \mathrm{I}_0 \exp[-\mu(E)\mathrm{T}(\mathbf{r}_{\perp})] \tag{2}$$

and

$$\phi(\mathbf{r}_{\perp}, E, \mathrm{R}_1) = -k\delta(E)\mathrm{T}(\mathbf{r}_{\perp}) \tag{3}$$

where, $\mu(E) = 2k\beta(E)$ is the linear attenuation coefficient and $\delta(E)$ is the decrement from unity of the of complex refractive index $n(E) = 1 - \delta(E) + i\beta(E)$ [3]. We emphasize that both quantities are dependent on the Energy E of the incident X-radiation. $\mathrm{T}(\mathbf{r}_{\perp})$ is projected thickness function of the object. I_0 is the incident intensity of the incoming beam. Substituting Eqs. 2 and 3 into Eqn. 1 then the left-hand-side of the resulting expressions appears as:

$$\frac{-k\mathrm{I}_0\delta(E)}{\mu(E)}\nabla_{\perp}^2 \exp[-\mu(E)\mathrm{T}(\mathbf{r}_{\perp})] \tag{4}$$

Now, the right-hand-side of Eqn. 1 can be approximated as the difference of two-closely spaced intensity images, namely $\mathrm{I}(\mathbf{r}_{\perp}, E, \mathrm{R}_2)$ and $\mathrm{I}(\mathbf{r}_{\perp}, E, \mathrm{R}_1)$ separated by the object-to-detector distance (ODD) R_2 as depicted in Fig 1. That is,

$$\frac{\partial}{\partial z}\mathrm{I}(\mathbf{r}_{\perp}, E, z) \approx \frac{\mathrm{I}(\mathbf{r}_{\perp}, E, \mathrm{R}_2) - \mathrm{I}(\mathbf{r}_{\perp}, E, \mathrm{R}_1)}{\mathrm{R}_2} \tag{5}$$

Substituting Eqn. 2 into Eqn. 5 then equating to Eqn. 4 the intensity at the detector plane ($z = \mathrm{R}_2$) for an arbitrary E is given by:

$$\mathrm{I}(\mathbf{r}_\perp, E, \mathrm{R}_2) = \mathrm{I}_0 \left[1 - \frac{\mathrm{R}_2 \delta(E)}{\mu(E)} \nabla_\perp^2\right] \exp[-\mu(E)\mathrm{T}(\mathbf{r}_\perp)] \tag{6}$$

One can make further simplifications by Taylor expanding the exponential term up to first order in $\mathrm{T}(\mathbf{r}_\perp)$ giving:

$$\mathrm{I}(\mathbf{r}_\perp, E, \mathrm{R}_2) \approx \mathrm{I}_0 + \mathrm{I}_0 \left[\mathrm{R}_2 \delta(E) - \mu(E) \nabla_\perp^2\right] \mathrm{T}(\mathbf{r}_\perp) \tag{7}$$

The differential equation has now become linear in $\mathrm{T}(\mathbf{r}_\perp)$ and is considerably simpler. Therefore, at this point it proves convenient to further generalize by taking accounting for polychromatic radiation. This is done by integrating the intensity $\mathrm{I}(\mathbf{r}_\perp, E, \mathrm{R}_2)$ at the detector plane over all possible incoming X-ray energies of the source spectrum. All energy spectrums of radiation sources, whether they are synchrotron, X-ray tubes, or free-electron lasers have an associated spectrum distribution function $h(E)$. This implies that every photon E has an associated statistical number of counts h [3]. Therefore, the intensity at the detector plane accounting for polychromatic radiation is given by the following integration:

$$\mathrm{I}^{\mathrm{Poly}}(\mathbf{r}_\perp, \mathrm{R}_2) = \frac{\int_{E_{min}}^{E_{max}} \mathrm{I}(\mathbf{r}_\perp, E, \mathrm{R}_2) h(E) dE}{\int_{E_{min}}^{E_{max}} h(E) dE} \tag{8}$$

Here, we have $E = E_{min}$ which is the lower bound corresponding to the lowest photon energy and E_{max} which is the upper bound corresponding to the highest photon energy. Notice that the expression has been normalized by the total number of counts which is a non-negative real valued constant $\bar{\gamma} > 0$, that is:

$$\int_{E_{min}}^{E_{max}} h(E) dE = \bar{\gamma} \tag{9}$$

Upon substitution of Eqn. (7) of into Eqn. (8) the integral involving the differential element dE only acts on $\delta(E)$ and $\mu(E)$ thus enabling these integrals to be separated. Hence, the expression for the intensity at $z = \mathrm{R}_2$ under polychromatic incident illumination still remains linear

$$\mathrm{I}^{\mathrm{Poly}}(\mathbf{r}_\perp, \mathrm{R}_2) = \mathrm{I}_0 + \mathrm{I}_0 \left[\mathrm{R}_2 \bar{\delta}_M - \bar{\mu}_M \nabla_\perp^2\right] \mathrm{T}(\mathbf{r}_\perp) \tag{10}$$

Here, it is important to make note that now that $\delta(E)$ and $\mu(E)$ have now become $\bar{\delta}_M$ and $\bar{\mu}_M$ as a result of separating the integrals which contain the variable E. In essence, these introduced terms are the weighted averages of $\delta(E)$ and $\mu(E)$ over the range of energies of the source spectrum of the beam. Also, like in Eqn. (9) these "weighted average" integrals give non-negative real valued constants as defines below:

$$\begin{aligned} \bar{\delta}_M &= \frac{\int_{E_{min}}^{E_{max}} \delta(E) h(E) dE}{\int_{E_{min}}^{E_{max}} h(E) dE} \\ \bar{\mu}_M &= \frac{\int_{E_{min}}^{E_{max}} \mu(E) h(E) dE}{\int_{E_{min}}^{E_{max}} h(E) dE} \end{aligned} \tag{11}$$

As a result one can now solve for $\mathrm{T}(\mathbf{r}_\perp)$ by Fourier transforming both sides of Eqn. (10) then making use of the Fourier derivative theorem to give

$$\mathrm{T}(\mathbf{r}_\perp) = \mathrm{F}^{-1}\left[\frac{1}{\mathrm{R}_2\bar{\delta}_M\mathbf{k}_\perp^2 + \bar{\mu}_M}\mathrm{F}\left\{1 - \frac{\mathrm{I}^{\mathrm{Poly}}(\mathbf{r}_\perp, \mathrm{R}_2)}{\mathrm{I}_0}\right\}\right] \tag{12}$$

where, F are F^{-1} are forward and inverse Fourier transforms, respectively. $\mathbf{k}_\perp = (k_x, k_y)$ are the transverse Fourier conjugate coordinates dual to $\mathbf{r}_\perp = (x, y)$. The following Fourier transform convention have been used:

$$G(\mathbf{k}_\perp) = \int g(\mathbf{r}_\perp)\exp(-2\pi i\mathbf{k}_\perp \cdot \mathbf{r}_\perp)d\mathbf{r}_\perp$$
$$g(\mathbf{r}_\perp) = \int G(\mathbf{k}_\perp)\exp(2\pi i\mathbf{k}_\perp \cdot \mathbf{r}_\perp)d\mathbf{k}_\perp \tag{13}$$

where, $G(\mathbf{k}_\perp) = \mathrm{F}\{g(\mathbf{r}_\perp)\}$. From here additional manipulations can be made to prove that one can arrive at exact form of Paganin *el al.* [1] original single-image phase-retrieval algorithm however this time including polychromatic effects. The left hand side of Eqn. (12) can be separated into two terms:

$$\mathrm{T}(\mathbf{r}_\perp) = \mathrm{F}^{-1}\left[\frac{1}{\mathrm{R}_2\bar{\delta}_M\mathbf{k}_\perp^2 + \bar{\mu}_M} \times \widehat{\delta}(\mathbf{k}_\perp)\right] - \mathrm{F}^{-1}\left[\frac{1}{\mathrm{R}_2\bar{\delta}_M\mathbf{k}_\perp^2 + \bar{\mu}_M}\mathrm{F}\left\{\frac{\mathrm{I}^{\mathrm{Poly}}(\mathbf{r}_\perp, \mathrm{R}_2)}{\mathrm{I}_0}\right\}\right] \tag{14}$$

where, $\widehat{\delta}(\mathbf{k}_\perp) = \mathrm{F}\{1\}$ is the Dirac delta. Using the Dirac delta sifting theorem the 1st term on the right hand side reduces to:

$$\mathrm{F}^{-1}\left[\frac{1}{\mathrm{R}_2\bar{\delta}_M\mathbf{k}_\perp^2 + \bar{\mu}_M} \times \widehat{\delta}(\mathbf{k}_\perp)\right] = \int \frac{\exp(2\pi i\mathbf{k}_\perp \cdot \mathbf{r}_\perp)}{\mathrm{R}_2\bar{\delta}_M\mathbf{k}_\perp^2 + \bar{\mu}_M}\widehat{\delta}(\mathbf{k}_\perp)d\mathbf{k}_\perp = \frac{1}{\bar{\mu}_M} \tag{15}$$

This makes Eqn. 14 become:

$$1 - \bar{\mu}_M\mathrm{T}(\mathbf{r}_\perp) = \mathrm{F}^{-1}\left[\frac{\bar{\mu}_M}{\mathrm{R}_2\bar{\delta}_M\mathbf{k}_\perp^2 + \bar{\mu}_M}\mathrm{F}\left\{\frac{\mathrm{I}^{\mathrm{Poly}}(\mathbf{r}_\perp, \mathrm{R}_2)}{\mathrm{I}_0}\right\}\right] \tag{16}$$

We recall the first order Taylor expansion Taylor expansion approximation of an exponential function.

$$1 - \bar{\mu}_M\mathrm{T}(\mathbf{r}_\perp) \approx \exp\left[-\bar{\mu}_M\mathrm{T}(\mathbf{r}_\perp)\right] \tag{17}$$

Making use of this approximation in "reverse" is the final step which leads to the proof that Paganin *et al.* [1] original form can be obtained and can also be applied to PBI images acquired with polychromatic radiation sources such as the one used to collect the data for this study. The original form being

$$\mathrm{T}(\mathbf{r}_\perp) = -\frac{1}{\bar{\mu}_M}\ln\left[\mathrm{F}^{-1}\frac{1}{\frac{\mathrm{R}_2\bar{\delta}_M}{\bar{\mu}_M}\mathbf{k}_\perp^2 + 1}\mathrm{F}\left\{\frac{\mathrm{I}^{\mathrm{Poly}}(\mathbf{r}_\perp, \mathrm{R}_2)}{\mathrm{I}_0}\right\}\right] \tag{18}$$

As intuitively predicted, this derivation reveals that all that is required to implement the method in the context of polychromaticity is to replace the values of δ and μ with $\bar{\delta}_M$ and $\bar{\mu}_M$.

The algorithm is a simple convolution of the normalized intensity image with the Fourier filter $\frac{1}{\alpha \mathbf{k}_\perp^2 + 1}$, where the value $\alpha = \frac{\mathrm{R}_2 \bar{\delta}_M}{\bar{\mu}_M}$ is known *a priori* and is the reason why the the method is stable under presence of noise. It is this feature which makes the algorithm practically advantageous even as image processing tool, which we do in this study by treating the collected PBI images as "unfiltered" radiographs (i.e. $\mathrm{I}^{\mathrm{Rad}} = \frac{\mathrm{I}^{\mathrm{Poly}}(\mathbf{r}_\perp, \mathrm{R}_2)}{\mathrm{I}_0}$) and the retrieved images as "filtered" radiographs (i.e. $\mathrm{I}^{\mathrm{Filt}} = \exp\left[-\bar{\mu}_M \mathrm{T}(\mathbf{r}_\perp)\right]$) as stated in Eqn (1) in the main manuscript.

References

1. Paganin D, Mayo SC, Gureyev TE, Miller PR, Wilkins SW. Simultaneous phase and amplitude extraction from a single defocused image of a homogeneous object. Journal of Microscopy. 2002;206:33–40.

2. Teague MR. Deterministic phase retrieval: a Green's function solution. Journal of the Optical Society of America. 1983;73:1434–1441.

3. Als-Nielsen Jens, McMorrow Des. In: Elements of modern X-ray physics. John Wiley & Sons, Inc.; 2011. p. 421.
